# Genitourinary Tuberculosis and the Potential Impact of Delayed Diagnosis in Europe

**DOI:** 10.5152/tud.2025.25017

**Published:** 2025-07-29

**Authors:** Guglielmo Mantica

**Affiliations:** Department of Surgical and Diagnostic Integrated Sciences (DISC), University of Genoa, Genoa, Italy

To the Editor, 

I am writing in response to the recent article titled “Urogenital Tuberculosis and Delayed Diagnosis: A Qualitative Study,” that analyzed the causes of delayed diagnosis of genitourinary tuberculosis (GUTB).^1^ I commend the authors for the approach of the study and appreciate its timely relevance, especially in light of the increasing incidence of GUTB in many European countries.

It is unfortunate that despite the critical findings of this and other research, awareness and knowledge about GUTB remain limited, especially within Europe.^2^ This shortfall is especially pronounced among general practitioners and urologists who are often the first line of defense in diagnosing such conditions. The article clearly highlights that a significant number of cases go undiagnosed due to the lack of clinical suspicion and the inadequacy of specific diagnostic tests for GUTB. In daily practice, for example, a patient presenting with sterile pyuria and lower urinary tract symptoms might undergo repeated empirical antibiotic treatments before tuberculosis is even considered, illustrating how subtle clinical clues may often be overlooked. These factors suggest an urgent need for enhanced education and training regarding this condition, as well as scoring systems to help clinicians, as for other diseases.^3^ For instance, a clinical checklist that flags patients with persistent urinary symptoms, sterile pyuria, and risk factors such as prior tuberculosis exposure may be useful in order to support clinicians in raising suspicion earlier.

As also pointed out in the study, many healthcare professionals may wrongly attribute GUTB symptoms to more prevalent maladies, leading to significant delays in diagnosis and treatment.^4^^,^^5^ This situation is compounded by a general lack of familiarity with the disease’s presentation and progression among medical staff. Increasing educational outreach and advocacy by scientific societies is essential to change this situation.

Implementing targeted training programs for healthcare providers, especially in primary care settings, can significantly improve the early identification of GUTB. These programs could focus on updating practitioners about the disease’s unique characteristics, necessary diagnostic protocols, and the importance of considering tuberculosis in differential diagnoses.

Furthermore, initiatives aimed at raising patient awareness are equally crucial. Patients are often unaware of GUTB and its potential ramifications, which could hinder their ability to communicate symptoms effectively to their healthcare providers. A greater emphasis on public health campaigns that educate communities about GUTB could lead to earlier consultation and reduce diagnostic delays.

In conclusion, it is imperative that both medical professionals and patients are well-informed about this condition to reduce diagnostic delays and improve patient outcomes. Collaboration among healthcare providers, public health authorities, and European scientific societies is necessary in order to address this growing health issue.

## Data Availability

The data that support the findings of this study are available upon request from the corresponding author.
